# 2699. Risk of Bacterial Bloodstream Infection from a Urinary Source Among Kidney Transplant Recipients

**DOI:** 10.1093/ofid/ofad500.2310

**Published:** 2023-11-27

**Authors:** Emily Eichenberger, Geeta Karadkhele, Aileen C Johnson, Stephanie M Pouch, Christian P Larsen

**Affiliations:** Emory School of Medicine, Atlanta, Georgia; Emory University School of Medicine, Atlanta, Georgia, Atlanta, Georgia; Emory School of Medicine, Atlanta, Georgia; Emory University School of Medicine, Atlanta, GA; Emory University School of Medicine, Atlanta, GA

## Abstract

**Background:**

Bacteriuria is common among kidney transplant recipients (KTR). Bloodstream infection is a feared complication of bacteriuria. Risk factors and outcomes associated with bloodstream infection due to a urinary source (BSIU) in KTR are poorly understood.

**Methods:**

In a single center retrospective review, we included all KTR at Emory Transplant Center who had bacteriuria between 06/2007-09/2022. BSIU included KTR with concordant positive blood and urine cultures. BN included KTR with bacteriuria who never developed a bloodstream infection (Figure 1). For subjects with more than one episode of BSIU or BN, only the first episode was included. Clinical characteristics were compared using Kruskal Wallis and fisher exact test. Multivariate logistic regression was used to determine factors associated with BSIU.

**Figure:**
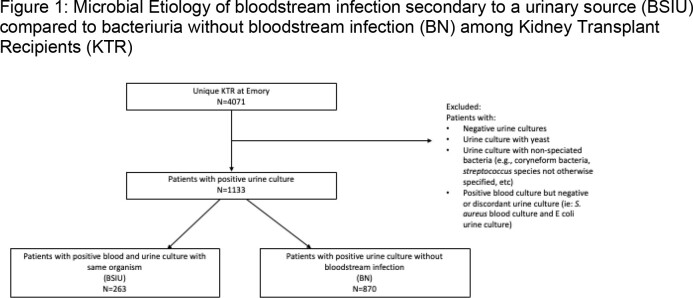

**Results:**

Among 4071 unique KTR, 1133 developed bacteriuria of which 263 (23.2%) were BSIU and 870 (76.8%) were BN (Table 1). KTR with BSIU were older (median age 54 vs 50, p=0.002) and more likely to have diabetes mellitus as the primary kidney disease (35.4% vs 23.2%, p=0.0004) than those with BN. Males comprised 49.4% of BSIU vs 30.8% of BN (p < 0.0001). Baseline renal function was significantly lower prior to BSIU than BN (glomerular filtration rate (GFR) 40.3 vs 46.8, p=0.0071). Enterobacterales caused 83.3% of BSIU vs 60.5% of BN, p < 0.0001 (Figure 2), of which 19.3% and 11.5% were extended spectrum beta-lactamase producing, respectively (p=0.007). After adjustment, male sex, decreased GFR, primary kidney disease secondary to diabetes mellitus, and presence of Enterobacterales were significantly associated with BSIU (Male: aOR 2.66 (95%CI 1.94, 3.64), diabetes aOR 1.86 (95%CI 1.33, 2.60), GFR aOR 0.99 (95% CI 0.99, 1.00), Enterobacterales aOR 3.55 (95%CI 2.23, 5.64), Table 2).

30-day mortality was 3% for BSIU vs 0.9% for BN (p=0.0168). Rate of acute cellular rejection at 90 days and graft failure 1-year post culture did not significantly differ between BSIU vs BN (rejection: 1.9% vs 3.6%, p=1.000; graft failure: 5.3% vs 3.1%, p=0.1292).

**Figure:**
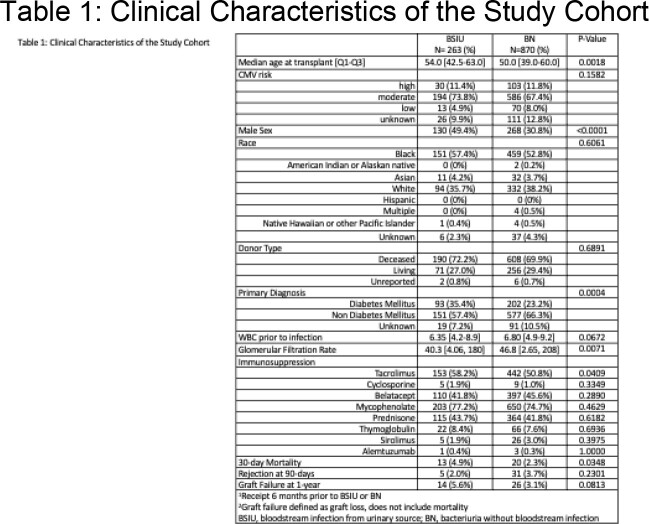

**Figure 2.**
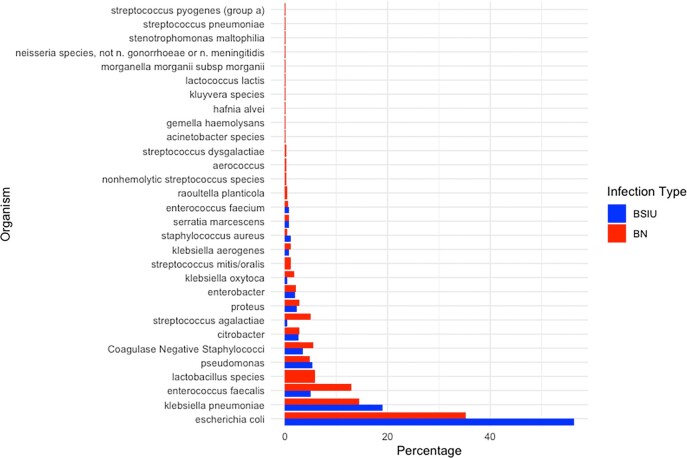
Microbial Etiology of bloodstream infection secondary to a urinary source (BSIU) compared to bacteriuria without bloodstream infection (BN) among Kidney Transplant Recipients

**Figure:**
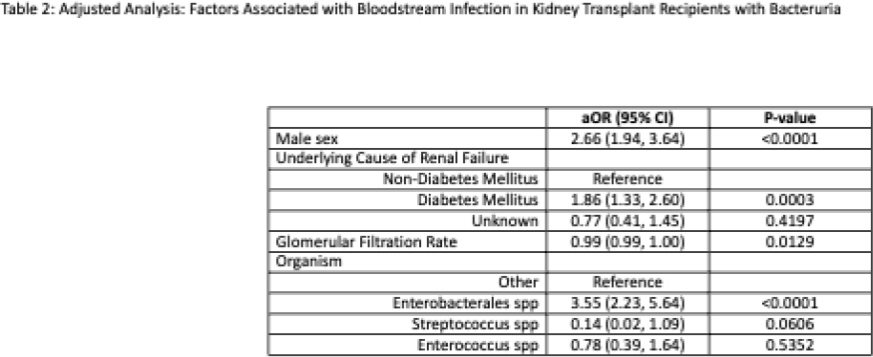

**Conclusion:**

Male sex, history of renal disease due to diabetes mellitus, decreased GFR and Enterobacterales were associated with BSIU among KTR. Mortality among BSIU was low, and there was no increased risk of graft failure or rejection relative to BN.

**Disclosures:**

**All Authors**: No reported disclosures

